# One Single Tube Reaction of Aptasensor-Based Magnetic Sensing System for Selective Fluorescent Detection of VEGF in Plasma

**DOI:** 10.3390/bios13060574

**Published:** 2023-05-24

**Authors:** Hwang-Shang Kou, Shao-Tsung Lo, Chun-Chi Wang

**Affiliations:** 1 School of Pharmacy, College of Pharmacy, Kaohsiung Medical University, Kaohsiung 807, Taiwan; 2 Department of Medical Research, Kaohsiung Medical University Hospital, Kaohsiung 807, Taiwan; 3 Drug Development and Value Creation Research Center, Kaohsiung Medical University, Kaohsiung 807, Taiwan

**Keywords:** VEGF, aptasensor, streptavidin magnetic bead, plasma, hybridization probe

## Abstract

In this study, a simple, easy and convenient fluorescent sensing system for the detection of the vascular endothelial growth factor (VEGF) based on VEGF aptamers, aptamer-complementary fluorescence-labeled probe and streptavidin magnetic beads was developed in one single tube. The VEGF is the most important biomarker in cancer, and it is investigated that the serum VEGF level varied according to the different types and courses of cancers. Hence, efficient quantification of VEGF is able to improve the accuracy of cancer diagnoses and the precision of disease surveillance. In this research, the VEGF aptamer was designed to be able to bind with the VEGF by forming G-quadruplex secondary structures; then, the magnetic beads would capture the non-binding aptamers due to non-steric interference; and finally, the fluorescence-labeled probes were hybridized with the aptamers captured by the magnetic beads. Therefore, the fluorescent intensity in the supernatant would specifically reflect the present VEGF. After an overall optimization, the optimal conditions for the detection of VEGF were as followed, KCl, 50 μM; pH 7.0; aptamer, 0.1 μM; and magnetic beads, 10 μL (4 μg/μL). The VEGF could be well quantified within a range of 0.2-2.0 ng/mL in plasma, and the calibration curve possessed a good linearity (y = 1.0391x + 0.5471, r = 0.998). The detection limit (LOD) was calculated to be 0.0445 ng/mL according to the formula (LOD = 3.3 × σ/S). The specificity of this method was also investigated under the appearance of many other serum proteins, and the data showed good specificity in this aptasensor-based magnetic sensing system. This strategy provided a simple, sensitive and selective biosensing platform for the detection of serum VEGF. Finally, it was expected that this detection technique can be used to promote more clinical applications.

## 1. Introduction

Over the past decade, the magnetic sensing system provided a convenient platform to simplify the process of the development of the sensing system, because the magnetic spherical materials could be easily applied to capture the targets and be simply collected by a magnet. Among them, streptavidin and biotin were often utilized in the magnetic sensing system due to their good specific binding [1], and the strong non-covalent binding force between streptavidin and biotin has been also widely used in many detection methods [2,3,4,5,6]. Additionally, the small-scale magnetic beads themselves also have a very high specific surface area, and thus, they have good dispersibility in solutions and the reaction rate with the target is very fast. The above advantages allow streptavidin-modified magnetic beads to be widely used and easily operated [7]. However, the magnetic beads could not supply the specific recognition of the targets. Therefore, several materials for specific binding with the targets were developed, such as traditional antibodies, aptamers, molecularly imprinted polymers [8] and so on. Among those, aptamers have the characteristics of low molecular weight, stability, easy modification, easy synthesis and not being affected by repeatability, and thus, in most of the current sensing methods, it has gradually replaced the role of the original antibody [9,10,11]. Aptamers refer to a sequence of about 20–60 nucleotides in length, which can be designed as specific binding with high affinity for different target molecules [12,13,14,15]. Under the binding reaction, the aptamers will be flexed into many various secondary structures, such as G-quadruplex which can specifically bind target molecules [16,17]. Therefore, aptamers are often applied in the development of biosensing systems [15,18,19,20]. In this study, the benefits of the magnetic sensing system and aptamers would be combined to develop an aptasensor-based magnetic sensing system for selectively fluorescent detection of vascular endothelial growth factor, VEGF, in plasma.

VEGF is a key factor in angiogenesis which is the formation and maintenance of blood vessel structures for physiological functions, and is considered an important component for the progression of diseases such as cancer and inflammation [21]. When tumor cells are attacked by immune mechanisms and poisonous drugs, they will develop drug resistance through their ability to randomly mutate in large numbers [22,23,24], making treatment even more difficult. Therefore, the direction of treatment began to shift to blocking tumor development and indirectly inhibiting tumor development, such as the blockage of angiogenesis [25,26,27]. According to the clinical literature, vascular endothelial cell growth factor (VEGF) in tumor cells would have a much different blood concentration from normal cells, and the angiogenesis rate of tumor cells is increased to twenty times as many as normal cells [28,29]. In the literature review of meta-analysis in 2007, it was mentioned that the blood concentration of VEGF in breast cancer patients was about 310 pg/mL, which was ten times as high as 30 pg/mL in normal human blood [30]. The blood concentration can be utilized to aid in the diagnosis of cancer types and also to monitor the progression of tumor cells throughout the course of the disease [31,32,33]. Therefore, rapid and accurate VEGF detection is particularly important.

Until now, a lot of techniques have been developed for the detection of VEGF for the current clinical applications, including traditional Western blot [34], immunohistochemistry (IHC) [35] and radioimmunoassay (RIA) [36]. At present, the enzyme-linked immunosorbent assay (ELISA) is the most commonly used clinical method for the detection of VEGF [37]. In addition, with the recent development of novel biosensing platforms, aptamer-based luminescence detection methods [38,39,40,41] were also widely developed. Other methods such as the colorimetric system [42], surface plasmon techniques [43], surface plasmon resonance imaging (SPRi) [44,45] have also been designed and applied. Among them, most of the methods required expensive testing materials and complex knowledge or operation to process and interpret the data. Therefore, in this study, a method using aptamers (APT), fluorescent probe and a simple magnetic bead was developed, which can be rapidly quantified under a fluorescent detector and completed in one single tube. This strategy provided a simple, sensitive and selective biosensing platform for the detection of serum VEGF, and it was expected that this detection technique could be severed as a tool for clinical survey, and promoted to more clinical applications in the detection of VEGR.

## 2. Materials and Methods

### 2.1. Reagents and Materials

VEGF-165 (VEGF, 100 µg/mL) were purchased from the Takapouzist Company (Tehran, Iran). VEGF-aptamer and fam-labeled probes were synthesized by MDBio Co., Ltd. (Taipei, Taiwan), the sequences of those were as shown in Table 1. Streptavidin magnetic beads (4 μg/mL, 1 µm) were purchased from New England Biolabs (Ipswich, MA, USA). Ethanol, 2,2′,2″,2‴-(Ethane-1,2-diyldinitrilo) tetraacetic acid (EDTA) and 2-Amino-2-hydroxymethyl-propane-1,3-diol (TRIS-base) were purchased from J.T.Bake^®^ (Phillipsburg, NJ, USA). The ddH_2_O was prepared by the Milli-Q^®^ system (Millipore, Bedford, MA, USA). Methanol (analytical grade reagent) and magnesium chloride were purchased from Merck (Merck, Darmstadt, Germany). Potassium chloride was purchased from Scharlau (Barcelona, Spain). Sodium chloride was purchased from PanReac AppliChem ITW reagents (Barcelona, Spain).

### 2.2. The Detection of the Fluorescence

The measurements of fluorescence were carried out in F-4500 fluorescent spectrometer (Hitachi, Tokyo, Japan). The excitation and emission slit were set at 10 nm and 10 nm, respectively. The scanning speed was set at 1200 nm/min. The fluorescence emission was recorded from 500 to 600 nm and the excitation wavelength was set at 480 nm. All measurements were carried out at room temperature.

The symbol of (F) meant the fluorescent intensity in the presence of different concentrations of the VEGF, and the (F0) meant the fluorescent intensity without VEGF. The final signal for method optimization, calibration curve, specificity and recovery would be expressed as shown in Equation (1).

(F − F0)/F0(1)

F: the fluorescent intensity in the presence of VEGF.
F0: the fluorescent intensity without VEGF.

### 2.3. The One Single Tube Reaction

Firstly, a total of 200 μL solution containing 50 μM KCl and 0.1 μM VEGF-aptamer was prepared in a 1X TBSE buffer (10 mM TRIS-Base, 0.05 mM EDTA, 100 mM NaCl and 1 mM MgCl_2_, pH 7.0). In order to allow the VEGF-aptamer to form the structure of the G-quadruplex, the above solution was heated to 95 °C for 3 min, and then cooled down to room temperature. Subsequently, 20 μL solution containing different concentrations of VEGF was added to the solution and incubated at room temperature (25 °C) with shaking for 45 min, and then, 10 μL of magnetic beads was added to capture the residual VEGF-aptamer at room temperature (25 °C) with shaking for 1 min. After that, the magnetic beads were collected by a magnet and the supernatant was removed, and then, the magnetic beads were washed with ddH_2_O three times. Finally, a 200 μL 1X TBSE buffer solution containing a 0.01 μM fluorescent probe was added to reconstitute magnetic beads with shaking for 3 min. After precipitating the magnetic beads, the supernatant was collected to measure the fluorescence. The VEGF could be easily quantified through the fluorescent intensity of the supernatant. In the whole procedure, all reactions were completed in one single tube.

### 2.4. Method Validation

In the establishment of the calibration curve, different concentrations of VEGF including 0.2, 0.5, 1.5, 2.0 ng/mL spiked into the plasma were utilized and proceeded in the one single tube reaction according to Section 2.3. The calibration curve was established by a comparison of the fluorescent signal ((F − F0)/F0) (Y axis) with the various concentrations of VEGF in ng/mL (X axis). In this study, the specificity of the method was investigated by using the proteins or polypeptides that are also presented in humans. In order to show a high degree of specificity of this strategy with using these common proteins or polypeptides, these contained 20 ng/mL bovine serum albumin (BSA), 20 ng/mL albumin (Albumin), 5 × 10^−3^ U/mL insulin (Insulin), 2 × 10^−3^ U/mL mL nitric oxide synthase (NOS). The 2 ng/mL VEGF as the control group using these common proteins or polypeptides was utilized to investigate the specificity according to the fluorescent signal ((F − F0)/F0).

### 2.5. The Real Plasma Samples

The plasma samples were directly analyzed under this technique. Under the optimal conditions for detection of VEGF (KCl, 50 μM; pH 7.0; aptamer, 0.1 μM and magnetic beads, 10 μL (4 ng/μL)), the different concentrations of VEGF were spiked at 0.2, 0.5, and 1.5, 2 ng/mL into the plasma to establish the calibration curve of real biological samples. The calibration curve was established by a comparison of the fluorescent signal ((F − F0)/F0) (Y axis) with the various concentrations of VEGF in ng/mL (X axis). Additionally, in order to know the quantitative efficiency of the strategy for the detection of plasma samples, the two different concentrations of VEGF (0.8 and 1.6 ng/mL) were spiked into the test plasma sample with a real concentration of 0.2 ng/mL, and then the concentrations of VEGF was calibrated according the established regression line in plasma to estimate the recovery of this method in plasma samples.

## 3. Result and Discussion

### 3.1. The Mechanism of One Single Tube Reaction of Aptasensor-Based Magnetic Sensing System

**Figure 1:**
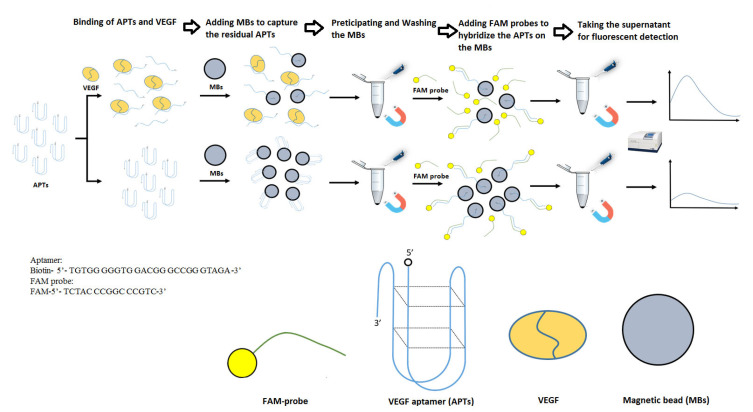
The mechanism of one single tube reaction of aptasensor-based magnetic sensing system for quantitatively sensing VEGF in plasma.

In this study, the one single tube reaction of the aptasensor-based magnetic sensing system was established for the detection of VEGF in plasma. The convenient, simple and fast sensing system presented that all of the detection could be completed in one single tube. The mechanism was as shown in Figure 1. Firstly, the APTs that have been flexed into the G-quadruplex in an appropriate environment are fully reacted with VEGF at room temperature for 40 min to generate a specific complex of non-covalent bonds between VEGF and the APTs G-quadruplex [46], and the residual APTs would retain the original G-quadruplex configuration in samples without VEGF. Subsequently, the streptavidin-coated magnetic bead (MB) was added into the solution to capture the residual APTs of the original G-quadruplex configuration through its derived biotin group, but the complex of APTs and VEGF would not be captured by the MBs due to the high steric interference. Therefore, more APT G-quadruplexes would be captured on the MBs in the absence of VEGF. After finishing that, the MBs were collected and purified with an external magnetic field, and the supernatant was removed. Finally, the MBs were washed with 1X TBSE buffer three times and reconstituted in a 1X TBSE buffer, and then, the fluorescent probe was added to the MBs whose surface is covered with some APTs. In the presence of VEGF, the residual APTs captured by the MB would be less, resulting in a lower amount of fluorescent probes that can bind to the MB through the hybridization. Therefore, the fluorescent intensity of the supernatant is higher. On the contrary, in the absence of VEGF, the number of residual APTs on the surface of the MBs would be larger, and a higher amount of fluorescent probes would be captured by the MBs, while the supernatant has a lower fluorescent intensity. Thus, in this simple design, the VEGF could be specifically quantified through the fluorescent intensity rebound ratio.

### 3.2. The Method Optimization

In this research, the aptasensor-based magnetic sensing system was developed in one single tube for the detection of VEGF in plasma. In order to obtain good sensitivity, specificity and stability under reactions, several parameters were optimized, including the concentration of KCl in the buffer, the buffer pH value (1X TBSE buffer), the concentration of APT, the length of fluorescent probe and the volume of the magnetic bead. The data and discussion were as shown in the following.

#### 3.2.1. The Concentration of KCl and the Buffer pH Value

**Figure 2:**
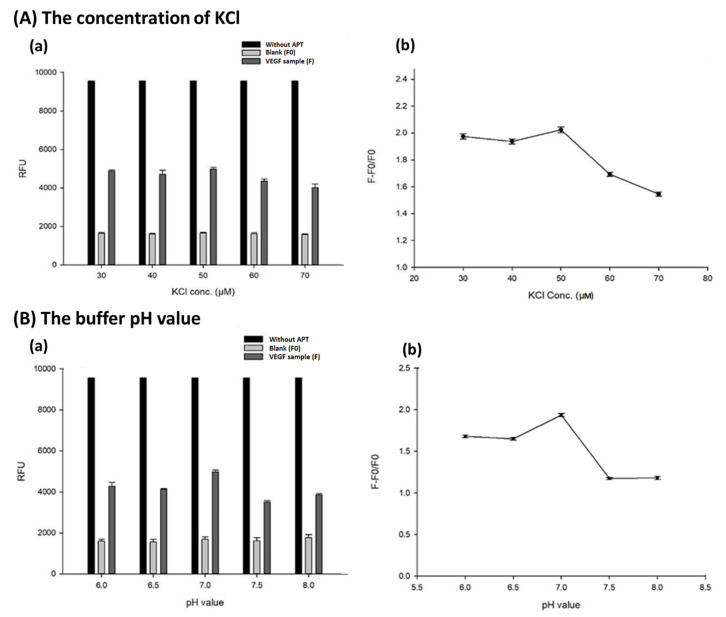
The effect of the concentration of KCl (**A**) and the buffer pH value (**B**) on the aptasensor-based magnetic sensing system. (**a**) The fluorescent intensity under various parameters (*n* = 3). Experimental conditions are as follows, pH = 7.0, VEGF conc., 2 ng/mL; magnetic beads (MB) volume, 10 μL (4 μg/mL); KCl conc., 50 μM; aptamer (APT) conc., 0.10 μM, fam-labeled probe length, 15 mer (0.01 μM). (**b**) The fluorescence ratios of F − F0/F0 under various conditions (*n* = 3).

According to the previous research [47,48], the concentration of KCl affects the formation of aptamers and the stability of the structure, which in turn, determines the affinity of aptamer with VEGF. However, excessively high electrolyte concentration will interact with the residues on the aptamer monomer through the ionic interaction, and electrostatic force acts to form undesired interactions and reduce the affinity of aptamers. Therefore, the evaluation of the concentration of KCl is a very important issue in the aptasensor-based sensing system. The different concentrations of KCl, including 30, 40, 50, 60 and 70 μM were investigated under the conditions of the volume of magnetic beads, 10 μL; the concentration of aptamer, 0.1 μM; the concentration of fluorescent probe, 0.01 μM; pH 7 and VEGF concentration of 2 ng/mL for specific detection. The data were as shown in Figure 2A. In Figure 2(Aa), the bar with the black color indicated the fluorescence without adding APT in the one single tube reaction. The data presented that the fluorescence without adding APT was stable and unified under various conditions. The bars with the light gray and deep gray color were blank and VEGF samples, respectively. From the data, the fluorescent intensities in the absence of VEGF (F0) and the presence of VEGF (F, 2 ng/mL) were significantly different. Therefore, from Figure 2(Ab), the fluorescence rebound ratio under different salt concentrations can be inferred, and in the case of the KCl concentration of 50 μM, the aptamer can form the configuration with the best binding effect to VEGF, and so the rebound ratio is the highest. Finally, the KCl concentration of 50 μM was selected as the optimal condition for this method.

The aptamer itself is a nucleotide sequence. Under the conditions of different pH values, the aptamer will pass through the amide group and the residue on the central carbon of the monomer to form different states of electricity. Therefore, the different pH values of the buffer would also affect the binding efficiency between the aptamer and the VEGF. In this research, different pH values of the 1X TBSE buffer were evaluated, including pH 6.0, 6.5, 7.0, 7.5 and 8.0. The data were as shown in Figure 2B. Since the aptamer can be regarded as a protein fragment with a complete tertiary structure, it is estimated that when the pH value is 7.4, close to the human body’s fluids, it will have a better affinity with VEGF. From the data of Figure 2(Ba), significantly different fluorescent intensities could be observed in the absence of VEGF (F0) and the presence of VEGF (F, 2 ng/mL) under different pH values. In Figure 2(Bb), when the pH of the aptamer is neutral (pH = 7.0), the binding effect of the aptamer to VEGF is the best, indicating that the aptamer can be bent into a high-affinity configuration in an environment close to the human body, so that the fluorescence rebound ratio reaches the maximum value within the range. Finally, pH 7.0 is selected as the best pH value for this method.

#### 3.2.2. The Concentration of APT and the Length of Fluorescent Probe

**Figure 3:**
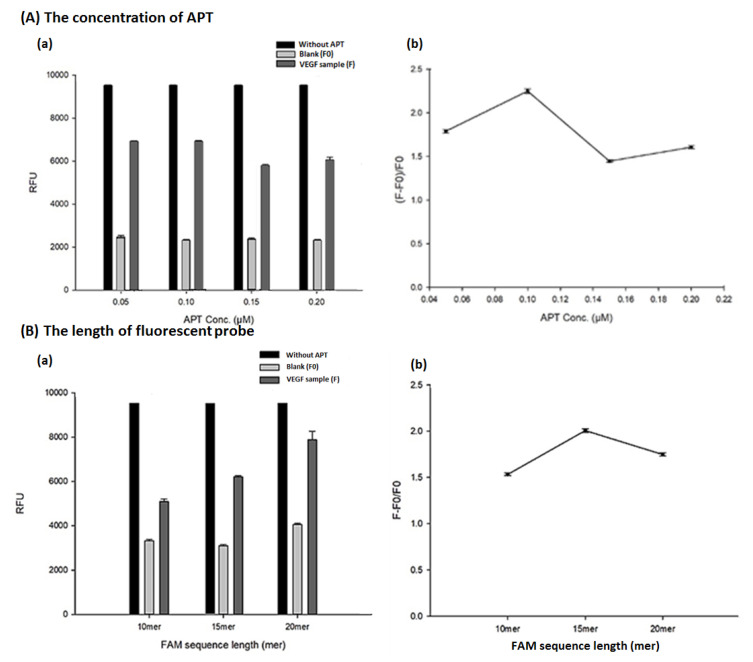
The effect of the concentration of APT (**A**) and the length of fluorescent probe (**B**) on the aptasensor-based magnetic sensing system. (**a**) The fluorescence under various conditions (*n* = 3). Experimental conditions are as follows, pH = 7.0, VEGF conc., 2 ng/mL; magnetic beads (MB) volume, 10 μL (4 μg/mL); KCl conc., 50 μM; aptamer (APT) conc., 0.10 μM, fam-labeled probe length, 15 mer (0.01 μM). (**b**) The fluorescence ratios of F − F0/F0 under various conditions (*n* = 3).

It is known that APTs can specifically capture the VEGF molecules by forming a structure flexed into a G-quadruplex through forces such as hydrogen bonds between base pairs on the sequence, and thus, different concentrations of APTs of 0.05, 0.10, 0.15, and 0.20 μM were evaluated. The data were as shown in Figure 3A. From Figure 3(Aa), higher concentrations of aptamers captured on the MB can hybridize more fluorescent probes resulting in the reduction of the fluorescence performance. Therefore, in the presence of VEGF samples, it was observed that the fluorescence decreased at higher APT concentrations. However, in the blank, the number of APTs at different concentrations was enough to saturate the MB, and there is no significant fluorescent difference between them. After using the F − F0/F0 fluorescence rebound (Figure 3(Ab), it was found that the maximum value occurred in the utilization of 0.1 μM APT. Therefore, through the evaluation of the ratio of fluorescence rebound, 0.1 μM APT was considered the best binding effect of VEGF in the data representation of aptamer concentrations, and selected as the optimal condition.

Because the length of the fluorescent probes would affect the binding strength of the APT captured on the MB, different lengths of the fluorescent probes, including 10 mer, 15 mer and 20 mer, were evaluated at the concentration of 0.01 μM. The data were as shown in Figure 3B. According to the principle of forming complementary hydrogen bonds between base pairs, it is estimated that when the fluorescent probe sequence is longer, the binding force with the APT on the MB should be higher. The phenome would result in a lower fluorescence being observed in the use of longer fluorescent probes. However, in Figure 3(Ba), the highest fluorescence took place in the use of the highest fluorescent probe (20 mer). The support of the longer probes would easily form the dimer structures that are difficult to hybridize with the APT, resulting in higher fluorescence in the longer probes. Through the evaluation of the amount of fluorescent rebound (Figure 3(Bb)), the fluorescent probe length of 15 mer had a good binding effect with the APT, and it is regarded as the optimization condition of this method.

#### 3.2.3. The Volume of Magnetic Bead (MBs)

**Figure 4:**
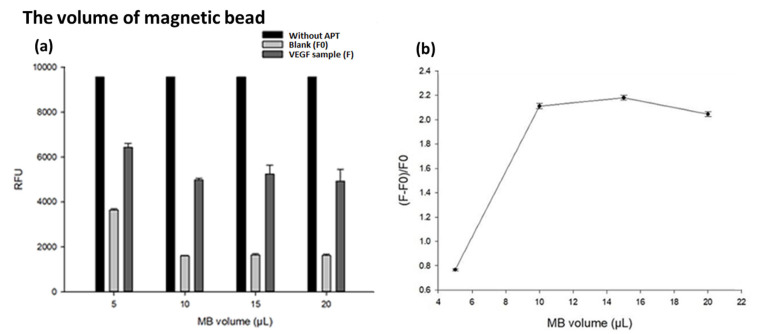
The effect of volume of magnetic bead on the aptasensor-based magnetic sensing system. (**a**) The fluorescence under various conditions (*n* = 3). Experimental conditions are as followed, pH= 7.0, VEGF conc., 2 ng/mL; magnetic beads (MB) volume, 10 μL (4 μg/mL); KCl conc., 50 μM; aptamer (APT) conc., 0.10 μM, fam-labeled probe length, 15 mer (0.01 μM). (**b**) The fluorescence ratios of F − F0/F0 under various conditions (*n* = 3).

Because the number of magnetic beads can fully carry the APT and the quantitative effect of the experiment will change due to the amount of MBs, different volumes of 5, 10, 15 and 20 μL (4 μg/mL) MBs were investigated. The data were as shown in Figure 4. The main function of magnetic beads is that they can bind with aptamers through covalent bonds and have a magnetic force. This magnetic system can help purify the target conjugates, and it can also be easily and quickly completed with magnets. In Figure 4a, with a volume of 5 μL MBs, the amount of MBs is not enough to carry all the aptamers, resulting in more free fluorescent probes and a higher fluorescence brightness. In the group with a volume of more than 10 μL, the amount of magnetic beads is enough to carry all the aptamers, so the capture effect for the fluorescent probe has reached saturation, resulting in similar fluorescence intensity. After referring to the rebound ratio (Figure 4b), the volume of magnetic beads of 10 μL is selected as the best and most economical choice.

### 3.3. The Specificity Test of the Aptasensor System

**Figure 5:**
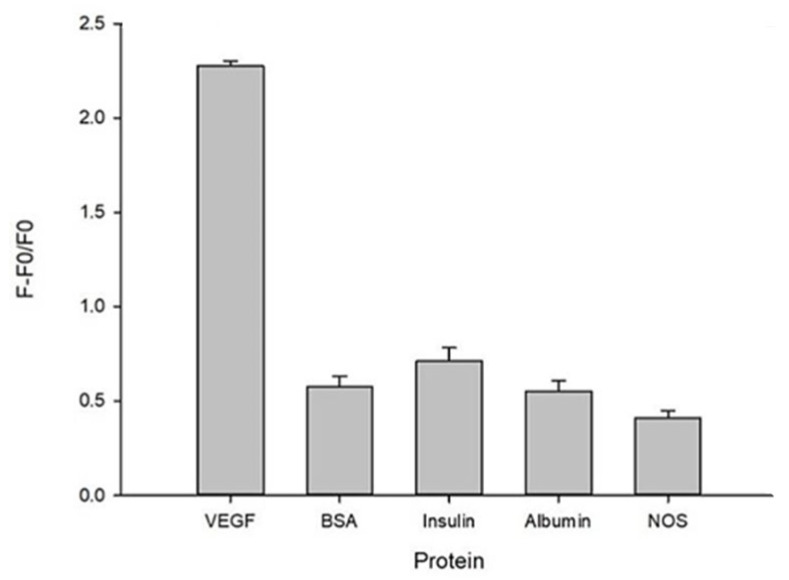
The specificity test of the aptasensor-based magnetic sensing system by using the fluorescence rebound ratio (F − F0/F0) in different kinds of regular proteins (*n* = 3). Experimental conditions are as followed, pH= 7.0, VEGF conc., 2 ng/mL; magnetic beads (MB) volume, 10 μL (4 μg/mL); KCl conc., 50 μM; aptamer (APT) conc., 0.10 μM, fam-labeled probe length, 15 mer (0.01 μM). VEGF conc.= 2.0 ng/mL, BSA conc. = 20 ng/mL, Albumin conc. = 20 ng/mL, insulin conc. = 5 × 10^−3^ U/mL, NOS = 2 × 10^−3^ U/mL.

In order to confirm that this aptasensor-based magnetic sensing system had a highly specific identification ability for VEGF and will not be affected by other similar proteins or polypeptides effects; thus, several substances commonly found in the human body were tested. Whether these common proteins or polypeptides can still maintain a high degree of specificity, these proteins or polypeptides contained 20 ng/mL bovine serum albumin (BSA), 20 ng/mL albumin (Albumin), 5 × 10^−3^ U/mL insulin (Insulin) and 2 × 10^−3^ U/mL mL nitric oxide synthase (NOS) which were used as the interfered proteins and VEGF 2 ng/mL as the control group to investigate the fluorescence rebound ratio (F − F0/F0). The data were as shown in Figure 5, and it can be confirmed that only the VEGF would have a significant fluorescence rebound ratio. The data demonstrated this aptasensor-based magnetic sensing system had high specificity when the concentration of other proteins is more than 20 times that which is presented in the human plasma, in comparison to the concentration of the test VEGF sample.

### 3.4. The Application for Real Plasma Samples

**Figure 6:**
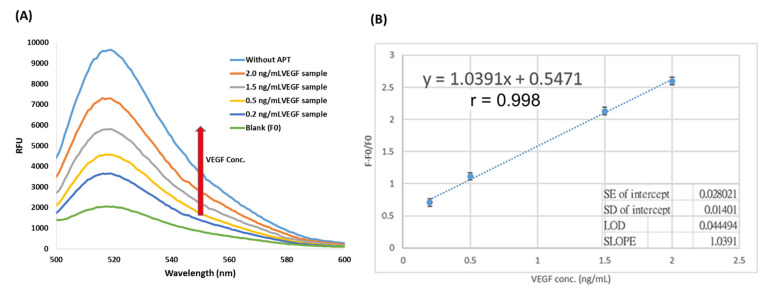
(**A**) The fluorescent spectra of the plasma samples spiked with different VEGF concentrations. (**B**) the calibration curve for quantification of VEGF by using F − F0/F0 vs the concentration of VEGF (*n* = 3). Experimental conditions are as followed, pH = 7.0, VEGF conc., 2 ng/mL; magnetic beads (MB) volume, 10 μL (4 μg/mL); KCl conc., 50 μM; aptamer (APT) conc., 0.10 μM, fam-labeled probe length, 15 mer (0.01 μM).

According to previous research [30], the VEGF concentrations in the plasma of cancer patients were about 2–10 times higher than those in healthy controls. The data were as shown in Table S1. Therefore, the establishment of the calibration curve for the quantification of VEGF in plasma was very important in clinical use. After method optimization, the optimal conditions for the detection of VEGF were as followed, KCl, 50 μM; pH 7.0; aptamer, 0.1 μM and magnetic beads, 10 μL (4 ng/μL). Under the optimal conditions, the various concentrations of VEGF from 0.1 to 10 ng/mL were used to establish the calibration curve (as shown in Figure S1). However, at the high level of the VEGF (5 ng/mL and 10 ng/mL), the binding of the APTs reached saturation, and the fluorescence was not changed. Therefore, the different concentrations of VEGF were spiked at 0.2, 0.5 and 1.5, 2 ng/mL into the plasma to establish the calibration curve of real plasma samples. The fluorescent spectra were as shown in Figure 6A. From the data, it could be found that the fluorescence rebound ratio (F − F0/F0) increased as the VEGF concentration increased. Additionally, without adding the APTs, the fluorescence was the highest one meaning the APTs could be effectively captured by the MBs, and then hybridize with the fluorescent probes to significantly reduce the fluorescence of the supernatant. When the VEGF was spiked into the plasma, the binding of APTs and VEGF would interfere with the MBs to capture the APTs, resulting in the rebound of the fluorescence. Therefore, the VEGF in plasma could be easily detected in one single tube reaction. According to the fluorescence rebound ratio versus the VEGF concentrations spiked into plasma, the calibration curve could be established as shown in Figure 6(B). The calibration curve possessed good linearity (y = 1.0391x + 0.5471, r = 0.998), and the detection limit (LOD) was calculated to be 0.0445 ng/mL, according to the formula (LOD = 3.3 × σ/S). After establishing the calibration curve, in order to know the recovery of this technique, two concentrations of 0.8 and 1.6 ng/mL VEGF were spiked into the known real plasma sample containing 0.2 ng/mL VEGF, respectively, and using the established regression line to infer the final concentrations. The data were as shown in Table 2. The recoveries of the two spiked concentrations were 116.71% and 102.52%, respectively, indicating that the sensing system can show a good detection effect in real samples.

## 4. Conclusions

In cancer treatment, the development of drugs has encountered a bottleneck, and clinical treatment policy has begun to shift to the biomarker of the proliferation of solid tumor cells, vascular endothelial cell growth factor (VEGF). In the review of meta-analysis in 2007, it was mentioned that the blood concentration of VEGF in breast cancer patients was about ten times that of normal human blood [30], so the value of the blood concentration of VEGF can not only be used to help the diagnosis of cancer types but also monitor the progress of tumor cells in treatment throughout the course of the disease. Therefore, the rapid and accurate detection of VEGF blood levels plays a very important role in cancer treatment. However, most of the current VEGF detection methods require professional detection instruments and experienced operators, and the detection process takes a relatively long time and is not universal [49]. Therefore, in this study, a simple, rapid and easy-to-use quantitative detection strategy, one single tube reaction of aptasensor-based magnetic sensing system has been successfully established for the detection of VEGF in plasma. This VEFG detection strategy developed by combining the design and optimized conditions of this study has been proven to be effective and specific in identifying VEGF in plasma, and all detection operations can be completed in as little as 50 min, which can achieve simple, fast, low-cost and high-sensitivity characteristics. This strategy provided a simple, sensitive and selective biosensing platform for the detection of serum VEGF, and it was expected that this detection technique can be used in more clinical applications.

## Figures and Tables

**Table 1:** The sequences of the VEGF aptamer and the fluorescent probes.

Label	Sequence	Length (mer)
VEGF aptamer	Biotin-5′-TGTGG GGGTG GACGG GCCGG GTAGA-3′	25
10 mer-probe	FAM-5′-TCTAC CCGGC-3′	10
15 mer-probe	FAM-5′-TCTAC CCGGC CCGTC-3′	15
20 mer-probe	FAM-5′-TCTAC CCGGC CCGTC CACCC-3′	20

FAM: 6-fluorescein amidite, fluorescent dye.

**Table 2:** Recovery values and precision of VEGF in plasma samples (*n* = 3).

Original VEGF (ng/mL)	Add VEGF (ng/mL)	Concentration Found (ng/mL)	Recovery (%)	RSD (%)
0.20	0.80	1.13 ± 0.03	116.71 ± 2.32	1.9
0.20	1.60	1.84 ± 0.06	102.50 ± 3.41	3.3

## Data Availability

Data reported in the study are available from the corresponding author on reasonable request.

## Supplementary Materials

The following supporting information can be downloaded at: https://www.mdpi.com/article/10.3390/bios13060574/s1, Figure S1: (A) The different concentrations of the VEGF versus the fluorescent intensities. (B) the calibration curve of VEGF by using F-F0/F0 vs the concentration of VEGF from 0.2 to 2.0 ng/mL; Table S1: The VEGF concentrations in the plasma of the healthy and cancer populations.
