# Supplementary material for: One Single Tube Reaction of Aptasensor-Based Magnetic Sensing System for Selective Fluorescent Detection of VEGF in Plasma
Source: Biosensors (Basel). 2023 May 24;13(6):574. doi: 10.3390/bios13060574 (PMC10296741; doi:10.3390/bios13060574)
Supplement: Supplementary file 1 [file biosensors-13-00574-s001.zip › biosensors-2373029-supplementary.pdf]

biosensors

MDPI

Supporting Information

One Single Tube Reaction of Aptasensor-Based Magnetic Sensing System for Selective Fluorescent Detection of VEGF in Plasma

Hwang-Shang Kou [1], Shao-Tsung Lo [1] and Chun-Chi Wang [1,2,3,*]

1 School of Pharmacy, College of Pharmacy, Kaohsiung Medical University, Kaohsiung, Taiwan
2 Department of Medical Research, Kaohsiung Medical University Hospital, Kaohsiung, Taiwan
3 Drug Development and Value Creation Research Center, Kaohsiung Medical University, Kaohsiung, Taiwan
* Correspondence: chunchi0716@kmu.edu.tw; Tel.: +886-7-3121101 (ext. 2253); Fax: +886-7-3210683

Table S1. The VEGF concentrations in the plasma of the healthy and cancer populations [30].

The Kind of Cancer	The Plasma VEGF of the "Healthy Individuals"	The Plasma VEGF of the "Cancer Patients"
Breast cancer	0.027 – 0.030 ng/mL	0.037 – 0.310 ng/mL
Prostate cancer	0.032 – 0.730 ng/mL	0.013 – 0.061 ng/mL
Colorectal cancer	0.019 – 0.211 ng/mL	0.009 – 0.126 ng/mL
Other cancer	0.023 – 0.137 ng/mL	0.009 – 0.026 ng/mL

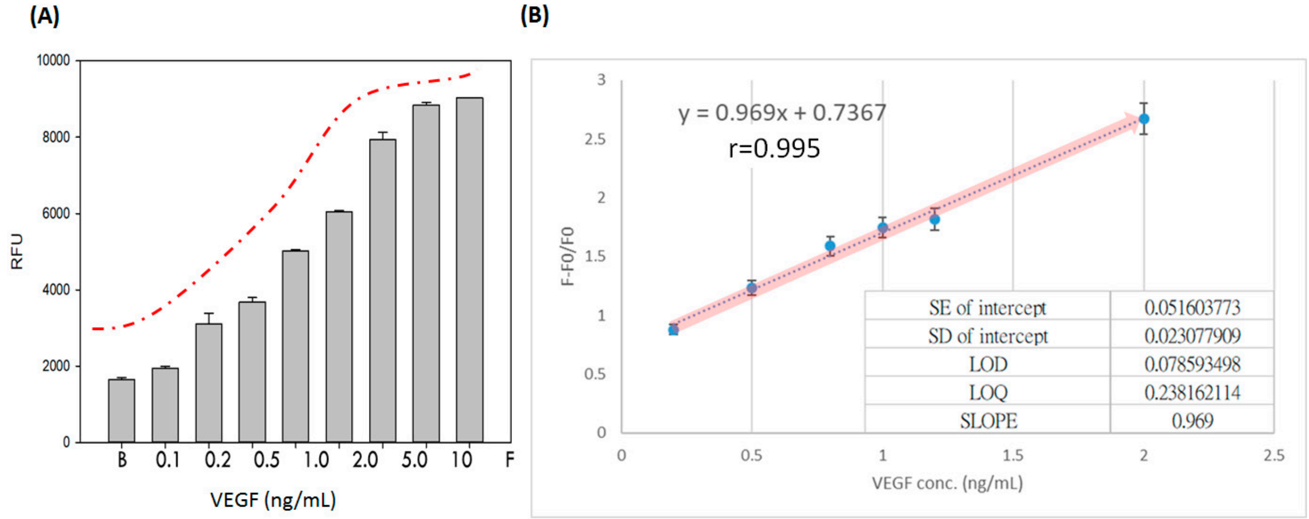

Figure S1. (A) The different concentrations of the VEGF versus the fluorescent intensities. (B) the calibration curve of VEGF by using F-F0/F0 vs the concentration of VEGF from 0.2 to 2.0 ng/mL.
